# Epidermal Grafting for Chronic Complex Wounds in India: A Case Series

**DOI:** 10.7759/cureus.516

**Published:** 2016-03-01

**Authors:** T.V. Prakash, Dr. Ajay Chaudhary, Shyam Purushothaman, Smitha K.V., Varada Arvind K.

**Affiliations:** 1 Community Health Centre, Naluthara, Pallor, Mahe, Union Territory of Pondichery; 2 Medicine, Acelity

**Keywords:** epidermal skin grafts, wound healing, chronic wounds

## Abstract

**Background:**

In India, the high cost of medical treatments and limited resources can deter patients from receiving available care, leading to the development of chronic wounds. We evaluated the use of epidermal grafting in patients with complex, long-term chronic wounds.

**Methods:**

Eighteen patients with complex wounds were treated with epidermal micrografts between September 2014 and March 2015 at a state-run, community health center in Mahe, Puducherry, India. Wound re-epithelialization was monitored for up to 14 weeks.

**Results:**

Comorbidities in the patient group (nine females and nine males; mean age 54.1 ± 10.8 years, range 32–70 years) included diabetes mellitus, hypertension, obesity (body mass index (BMI) >30 kg/m^2^), and peripheral vascular disease. The wound types included diabetic and nondiabetic foot, pressure, and venous leg ulcers. The average wound age prior to treatment was 36.8 ± 48.5 months (range 2–180 months) in the majority of patients. All wounds measured less than 7 cm × 7 cm. The mean time to wound epithelialization was 3.7 ± 1.8 weeks (range 2–9 weeks). The majority of wounds healed following epidermal grafting (n=16, 88.9%). One patient developed infection following removal of the dressing under non-sterile conditions against the advice of the healthcare providers. Another patient developed wound hypergranulation after grafting. Both wounds healed completely after treatment with antibiotic therapy and tissue resection, respectively. All donor sites healed without complications.

**Conclusion:**

In patients with small- to medium-sized chronic wounds, epidermal grafting offered a viable wound closure option for wounds requiring only the epidermal layer. Additionally, epidermal grafting was performed in the clinic without anesthesia or a surgeon, making the procedure more accessible in resource-challenged regions.

## Introduction

Healthcare costs have been steadily increasing worldwide. In India, public healthcare funding has been reported at 5% of the annual gross domestic product, with a majority (approximately 80%) of healthcare costs met from out-of-pocket payments [1]. High costs of treatment can deter patients from seeking care, potentially leading to the development of complex or chronic wounds, compounded by poor access to healthcare, lack of adequate manpower, and inadequate healthcare infrastructure [2]. An additional factor in the development of a chronic wound is underlying comorbidities, such as diabetes mellitus and peripheral vascular disease (PVD), which alter the wound healing process. In 2014, the prevalence of diabetes mellitus in India had increased to 9.1% and is expected to continue rising [3]. Diabetes affects wound healing through the loss of protective sensations due to neuropathy, development of ischemia, decreased inflammatory responses, and increased risk of infection [4-5]. PVD can cause morbidity and mortality, especially in elderly and diabetic populations [6]. The risk of PVD increases with age and diabetes diagnosis [6-7] and affects wound healing through neuropathy and reduced blood and oxygen flow to the affected area [8-9]. These underlying patient comorbidities complicate wound treatment and contribute to the development of chronic wounds.

A 2005 community-based epidemiological study identified the prevalence of chronic wounds in India to be 4.5 per 1000 of the population, with lower extremity wounds being the most common [2]. Untreated or inadequately treated acute traumatic wounds are a frequent cause of these chronic wounds [2,10]. Chronic wounds may remain unresponsive to conventional wound care treatments (topical agents and/or wound dressings), and skin grafts can be used for primary closure. Split-thickness skin grafting is a widely used procedure; however, it has some disadvantages. Split-thickness skin grafts (STSGs), obtained through a surgical procedure in an operating room under anesthesia, create not only a second wound at the donor site but also increase the costs of wound care. Complications during STSG healing, including graft contraction at the recipient site, can worsen patient morbidity and alter the patient’s quality of life [11-12]. However, another skin grafting option—epidermal grafting—can be performed in an outpatient setting with minimal pain and either limited or no scarring at the donor site.

Epidermal skin grafting can offer healthcare providers an alternative to split-thickness grafting when only the epidermal skin layer is required for wound closure. Introduced in the 1960s, epidermal skin grafting used suction to raise the epidermal skin layer, which was removed with a scalpel [13-14]. Recently, an automated epidermal harvesting system has become commercially available that utilizes negative pressure and heat to raise the epidermal skin layer, allowing for consistent and reproducible epidermal harvesting. This procedure can be performed in the physician’s office or at the patient’s bedside without anesthesia. We evaluated the use of the automated epidermal harvesting system to harvest epidermal micrografts for use over small- to medium-sized complex, nonhealing wounds for closure by tertiary intent.

## Materials and methods

### Patients

We conducted a retrospective review of medical records from patients with complex, nonhealing wounds who were treated with epidermal micrografts between September 2014 and March 2015 at a state-run, 30-bed community health center in Mahe, Puducherry, India. All patients provided consent for the procedure.

### Epidermal harvesting

Most wounds (13/15) were treated with negative pressure wound therapy (NPWT; V.A.C.^®^ Negative Pressure Wound Therapy, KCI, an Acelity company, San Antonio, TX) to promote healthy granulation tissue formation. Donor sites with healthy skin were selected, prepared by hair removal, and washed with 70% isopropyl alcohol prior to use of the epidermal harvesting system (CELLUTOME™ Epidermal Harvesting System; KCI, an Acelity company, San Antonio, TX). A sterile single-use harvester, was attached to the donor site, and the vacuum head on the harvester applied negative pressure (−400 to −500 mmHg) and warmth (37–41°C) to induce epidermal microdome formation (20–45 minutes). All microdomes were transferred onto a transparent polyurethane dressing (Tegaderm™ Transparent Film Dressing, 3M India Limited, Bangalore, India), which was then fenestrated with a sterile 18G needle and applied over the wound. The graft site was covered with a bolster dressing consisting of sterile gauze. The donor site was covered with gauze or transparent polyurethane dressing. The patients received a course of prophylactic antibiotics. Wound re-epithelialization was monitored at each dressing change and at weekly follow-up visits for up to 14 weeks. The wounds were considered healed when complete closure with full re-epithelialization was observed by the physician. 

### Statistics

Descriptive statistics were described as means, standard deviations, minimums and maximums for continuous variables, and frequencies and percents for categorical variables.

## Results

### Patient demographics

Between September 2014 and March 2015, 18 patients with small- to medium-sized complex, nonhealing wounds were treated with epidermal micrografts. This case series included nine females and nine males (mean age 54.1 years ± SD 10.8 years; range 32–70 years) (Table 1). The most common comorbidity in this patient group was diabetes mellitus (14/18, 77.8%), followed by hypertension (4/18, 22.2%), obesity (body mass index, BMI, >30kg/m^2^, 3/18, 16.7%), PVD (2/18, 11.1%), past tobacco use (2/18, 11.1%), active tobacco use (1/18, 5.6%), and rheumatoid arthritis (1/18, 5.6%).

**Table 1:** Patient demographics. sd = standard deviation.

Characteristic	N = 18; (n%)
Age (years)	
Mean (sd)	54.1 (10.8)
Range (years)	32-70
Sex	
Female	9 (50%)
Male	9 (50%)
Comorbidities	
Diabetes mellitus	14 (77.8%)
Hypertension	4 (22.2%)
Obese (BMI>30kg/m^2^)	3 (16.7%)
Peripheral vascular disease	2 (11.1%)
Tobacco use	
Past tobacco use	2 (11.1%)
Active tobacco use	1 (5.6%)
Rheumatoid arthritis	1 (5.6%)

The wound characteristics are presented in Table 2. The wound age prior to treatment was unknown in one patient. Of the remaining 17 patients, the average wound age was 36.8 ± 48.5 months (range 2–180 months). A majority of patients presented with a single wound (16/18, 88.9%) and the most common wound type presented was diabetic foot ulcer (12/18, 66.7%). Other wound types included nondiabetic foot ulcer (4/18, 22.2%), pressure ulcer (1/18, 5.6%), and venous leg ulcer (1/18, 5.6%). Previous treatments included debridement, antibiotic therapy, standard wound care dressings, and previous STSGs.

**Table 2:** Wound types. *One paitent was lost to follow-up; sd = standard deviation.

Characteristic	N = 18; n (%)
Wound duration (months) (n=17)*	
Mean (sd)	36.8 (48.5)
Range (months)	2-180
Number of wounds	
1	16 (88.9%)
2	2 (11.1%)
Wound type	
Diabetic foot ulcer	12 (66.7%)
Non-diabetic foot ulcer	4 (22.2%)
Pressure ulcer	1 (5.6%)
Venous leg ulcer	1 (5.6%)

### Graft outcomes

Wounds measuring 7 cm × 7 cm or smaller were identified for epidermal grafting. Highly exudative wounds, wound larger than 7 cm × 7 cm, and wounds without healthy granulation tissue or with signs of infection were excluded from treatment. The patients with poor glycemic control or unhealthy skin over the donor site (medial aspect of the thigh or upper arm) were not considered suitable for epidermal grafting and were excluded.

The mean time to epithelialization was 3.7 ± 1.8 weeks (range 2–9 weeks) (Table 3). The majority of wounds healed following epidermal grafting (16/18, 88.9%). Two patients showed delayed healing, secondary to complications: one non-compliant patient, who removed the dressing under non-sterile conditions, developed an infection that resolved with antibiotic therapy, allowing the wound to heal completely. The other patient developed hypergranulation at the wound site following epidermal grafting. Resection of the excessive granulation tissue enabled the wound to heal completely by week seven. All patients reported minimal or no pain at the donor site with no hemorrhagic or defective blisters and no bruising. All donor sites healed without complications.

**Table 3:** Outcomes of graft application. sd = standard deviation.

Characteristic	N = 18; n (%)
Time to epithelialization (weeks)	
Mean (sd)	3.7 (1.8)
Range (weeks)	2-9
Outcome of wound	
Healed	16 (88.9%)
Delayed healing	2 (11.1%)
Outcome of donor site	
Healed	18 (100%)
Not healed	0 (0%)

### Representative cases

Case 1

**Figure 1:**
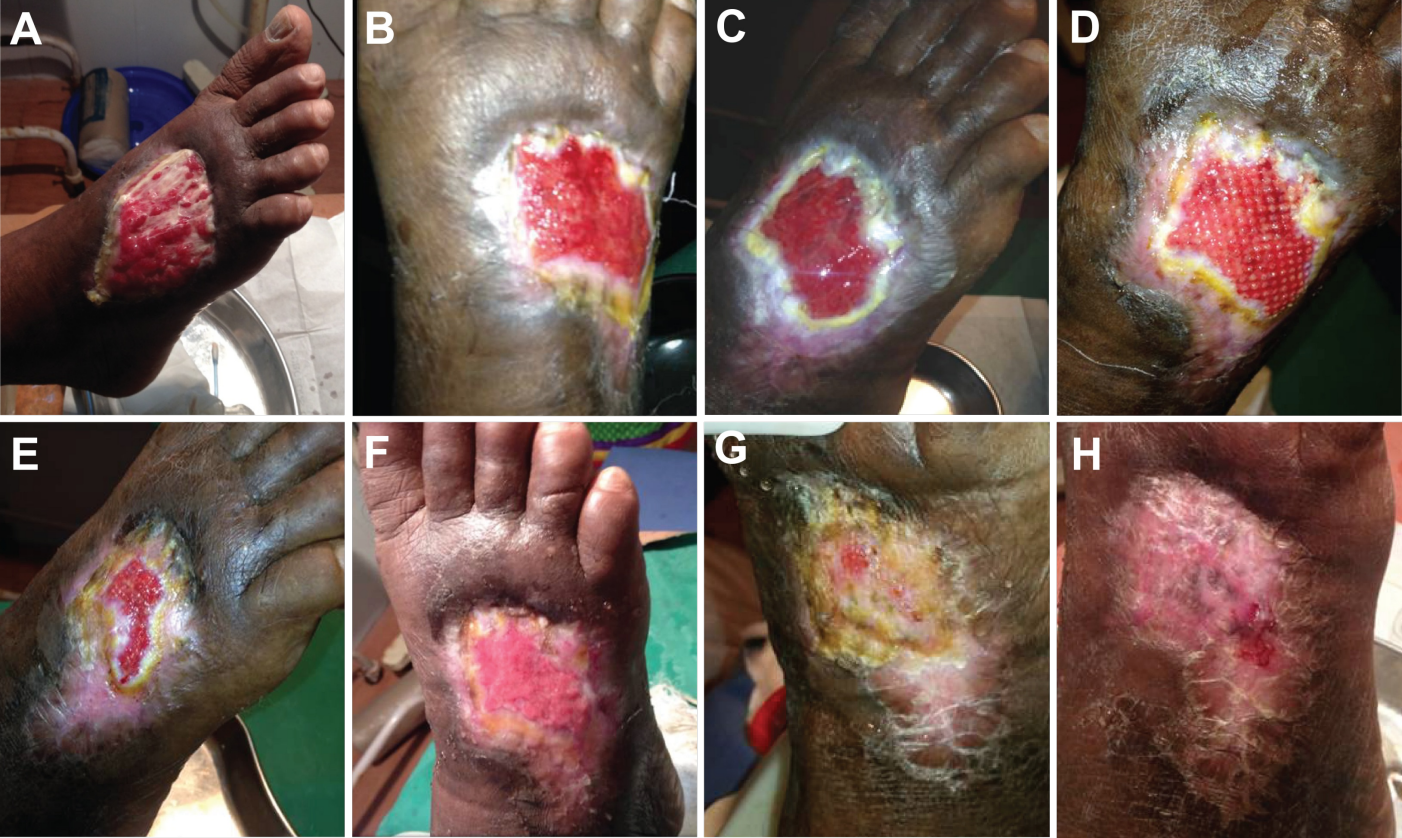
Epidermal grafting in a diabetic foot ulcer previously unresponsive to two years of treatment with standard wound care dressings. A. Wound at presentation. B. Epidermal micrograft application. C. Wound at seven days post grafting. D. Micrograft islands observed at 14 days post grafting. E. Wound re-epithelialization at 20 days post grafting. F. Wound at 27 days post grafting. G. Wound at 34 days post grafting. H. Wound completely healed at 40 days post grafting.

A 62-year-old male with a history of diabetes mellitus and active tobacco use presented with a diabetic foot ulcer (measuring 5 cm × 6 cm), nonhealing for two years, on the dorsum of the right foot (Figure 1A). Previous treatment involved standard wound-care dressings as well as antidiabetic therapy. At the time of grafting, the wound bed was well granulated, with evident vascularity, and minimal slough that was debrided before graft placement (Figure 1B). The patient received one application of epidermal micrografts harvested from the left thigh along with prophylactic oral antibiotics. The donor site healed uneventfully within a few days, and the wound healed completely without complication 40 days post grafting (Figure 1C, 1D, 1E, 1F, 1G, 1H).

Case 2

**Figure 2:**
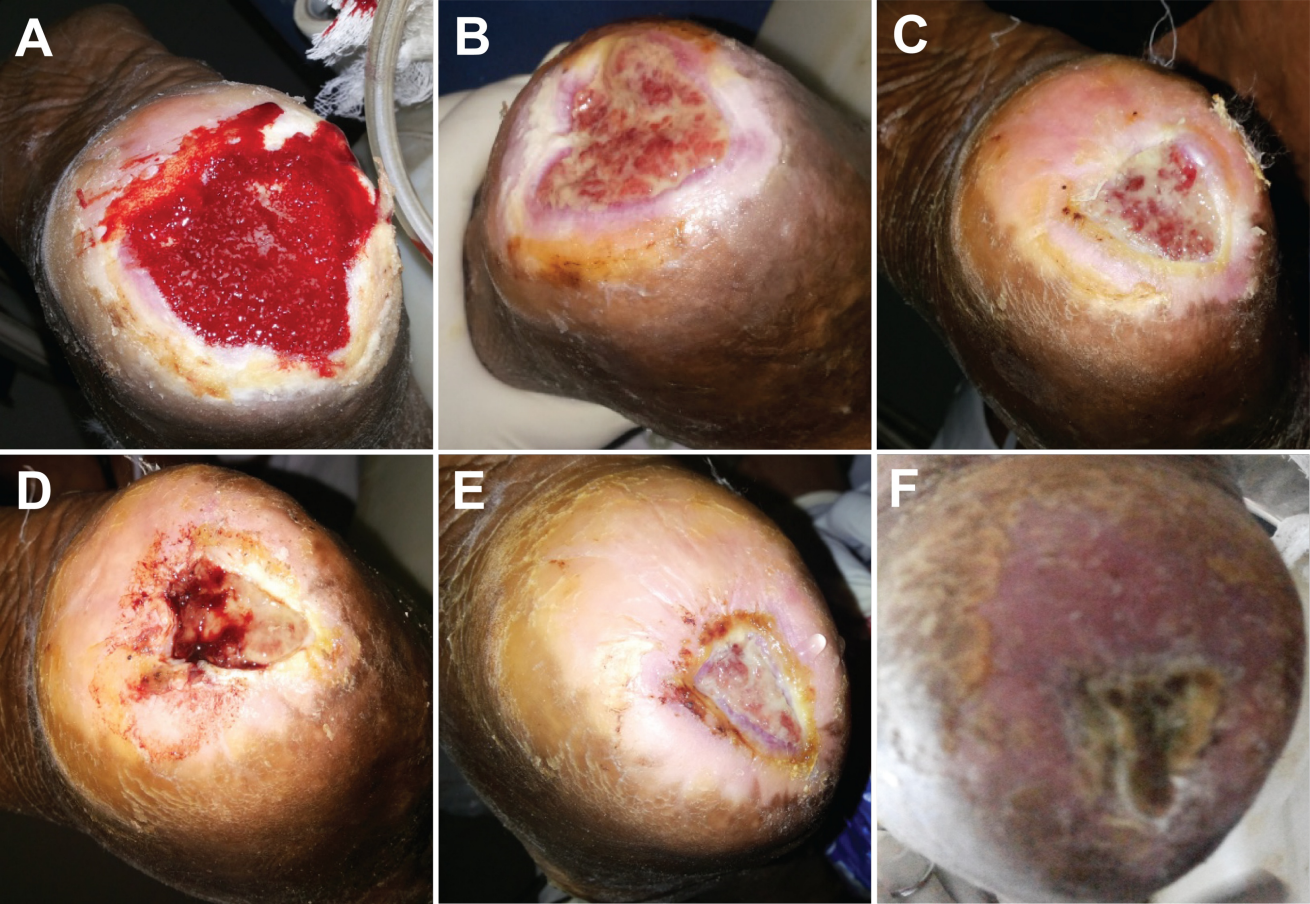
Epidermal graft use in a three-month-old pressure ulcer on the right heel in a paraplegic patient. A. Wound following seven days of treatment with NPWT. B. Wound at seven days post grafting. C. Wound at 18 days post grafting. D. Wound at 25 days post grafting. E. Wound at 31 days post grafting. F. Wound healed at 59 days post grafting.

**Figure 3:**
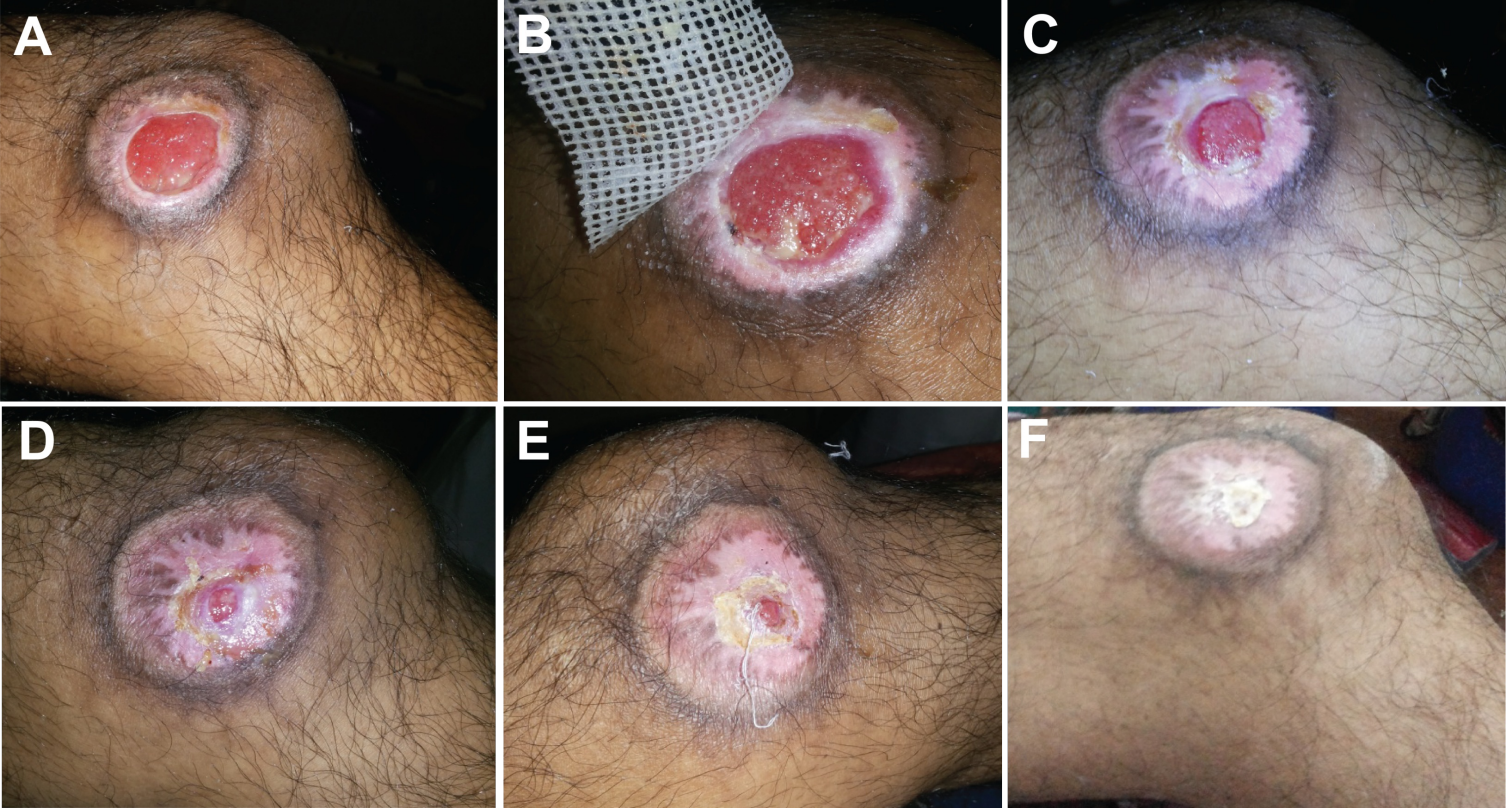
Epidermal graft use in a three-month-old pressure ulcer on the left knee in a paraplegic patient. A. Wound at presentation. B. Wound at seven days post grafting. C. Re-epithelialization observed at 18 days post grafting. D. Wound at 25 days post grafting. E. Wound at 31 days post grafting. F. Wound completely healed at 59 days post grafting.

A 35-year-old male with a history of paraplegia presented with a pressure ulcer on the right heel (5 cm × 4.5 cm) and an ulcer on the left knee (3 cm × 2.5 cm), both of three months duration. Previous treatments included oral antibiotics and standard wound care dressings. Prior to epidermal grafting, the heel wound underwent NPWT set at –125 mmHg in continuous mode for the first day and then in intermittent mode for six days with one dressing change. Epidermal grafts were applied on both wounds from a single epidermal graft taken from the left thigh and divided between the two wounds. A course of prophylactic oral antibiotics was given. The wound bed was prepared and had healthy granulation tissue suitable for graft take in both wounds; wound epithelialization was observed by four weeks following epidermal grafting. The pressure ulcer on the right heel (Figure 2), the ulcer on the left knee (Figure 3), and the donor site healed without complications.

## Discussion

Grafting with autologous tissues is a preferred treatment for certain difficult-to-treat chronic wounds. The use of STSG for such wounds is limited by the requirement for anesthesia, a functional operating room, and a skilled surgeon well versed in grafting. Additional disadvantages include donor site pain and complications, as well as patient-related considerations. At a relatively small health center such as ours, we encounter many of these limitations in addition to the economic constraints of our patient populations. Epidermal grafting seems compatible with a spectrum of wounds for which STSGs may be indicated. Here, we evaluated the use of an automated epidermal harvesting system to carry out epidermal grafting in 18 patients with complex, nonhealing wounds.

The majority of the wounds healed uneventfully following epidermal grafting (88.9%). Two patients developed complications (infection and hypergranulation) that resolved following appropriate treatment, and both wounds healed completely. The majority of wounds were prepared with NPWT to promote the development of healthy granulation tissue to prepare the wound for graft take. However, previous studies examining the use of epidermal grafting in chronic wounds have reported similar rates of wound healing as that seen in our cohort. In the Gabriel study [15], three of the four wounds treated with epidermal grafting completely healed. One wound (a diabetic foot ulcer present for eight years) showed 50% wound reduction at the end of the two-month follow-up period [15]. In the Richmond study [16], all five patients previously had been diagnosed with pyoderma gangrenosum that caused chronic, recurrent skin ulcers; however, all wounds achieved either complete healing or wound size reduction by eight weeks after epidermal grafting [16]. The Serena cohort [17] consisted of seven patients in Haiti with longstanding wounds. Six patients achieved complete wound closure four weeks following epidermal grafting [17]. In all of these studies, including our own cohort, the donor site healed completely without complications [15-17]. During the epidermal harvesting procedure, minimal to no pain was reported by our patients. Bruising or scarring of the donor site were not observed in our patients, which was most likely due to harvesting only the epidermal layer and leaving the dermal layer untouched and intact.

Based on our experience, the prerequisites for successful epidermal grafting are appropriate wound selection, wound preparation, and post-grafting care. Healthy small- to medium-sized wounds with granulation tissue and no necrotic tissue or slough are optimal wounds for epidermal grafting. It is equally important that the donor site consists of healthy, scar-free skin. Adequate wound preparation using adjunctive therapies, such as NPWT, can promote granulation tissue formation, improving the wound environment prior to grafting. Suitable post-grafting care includes use of nonadherent dressings for wound coverage, exudate management, and wound cleansing with saline. While epidermal grafts are proliferating, it is better to avoid wound debridement and use of antiseptic agents/wound irrigation solutions that may be toxic to healthy tissue. Antibiotic treatment may be initiated in patients at risk of developing infections.

The success rate of graft take in STSGs ranges from 85% to 100%, depending on the recipient wound bed characteristics [18-20]. In our small case series, we found epidermal graft take and healing to be comparable to rates published for STSGs. Although extrapolation of the results of this study is limited by the small number of cases, the results suggest the viability of epidermal grafting as a wound closure option in complex, chronic wounds.

Limited evidence exists regarding the mechanism of action for epidermal grafting. It is believed that wound closure occurs through keratinocyte migration and proliferation outward from the epithelial edge. Secretion of growth factors further drives cell migration until the entire wound area has been re-epithelialized [21-23]. A healthy human study (n=15) by Osborne and colleagues [24-25] examined the characteristics of epidermal skin grafts harvested using the automated epidermal harvesting system. The epidermal micrografts harvested from 12 patients exhibited almost complete viability (average 99.5%) [24]. Micrografts from three patients contained both keratinocytes and melanocytes that were able to proliferate in culture and actively secrete growth factors [25].

In this same healthy human study, the 15 healthy individuals reported minimal to no pain during the epidermal harvesting procedure [25]. In contrast, STSGs require the removal of the epidermal layer and a variable thickness dermis layer, which is performed in an operating room under anesthesia and creates a secondary wound at the donor site [26]. The STSG harvesting procedure creates donor site wounds that can be painful and difficult to heal [27-28]. Initial donor site pain following graft harvesting is often rated higher on the pain scale (indicated as more severe) than the pain from the graft recipient site [27-28], most likely due to the invasiveness of the procedure and the removal of skin structures. In our study, minimal to no pain was reported during or after the epidermal graft harvesting procedure. Complications in wound healing can lead to graft contraction which, depending on the location, may alter the function of surrounding skin and tissues [11]. Complications in donor-site wound healing can lead to scarring [26]. In our study, most of the epidermal grafts appeared to have taken on the physical characteristics of the recipient site, matching the epidermal grafted area and surrounding tissue with regard to color and texture, resulting in good aesthetic outcomes. As there is no graft shrinkage with epidermal grafting, a more uniform surface texture was attained in the healed wounds. 

## Conclusions

In this retrospective study, we observed successful wound closure following epidermal grafting in 18 patients with small- to medium-sized complex, chronic wounds that did not respond to conventional wound treatment. Epidermal grafting can be a suitable option for wounds missing the epidermal layer that require tertiary intent closure when STSG may not be a feasible option due to patient and cost factors. Additionally, epidermal grafting can be performed in the clinic or at patient bedside without anesthesia or a surgeon, thus making the procedure more accessible in resource-challenged areas.
